# Analysis of Microstructure Evolution and Mechanical Properties during Compression of Open-Cell Ni-Foams with Hollow Struts Using Micro-CT and FEM

**DOI:** 10.3390/ma15010124

**Published:** 2021-12-24

**Authors:** Jun Ho Lee, Geon Young Lee, Jong-joo Rha, Ji Hoon Kim, Jae-Hyung Cho

**Affiliations:** 1 Korea Institute of Materials Science, 797 Changwondaero, Seongsan-gu, Changwon, Gyeongnam 51508, Korea; f0020@naver.com (J.H.L.); arebee@kims.re.kr (G.Y.L.); jjrha@kims.re.kr (J.-j.R.); 2 School of Mechanical Engineering, Pusan National University, 2 Busandaehak-ro 63beon-gil, Geumjeong-gu, Busan 46241, Korea; kimjh@pusan.ac.kr

**Keywords:** Ni foams, micro-CT, 3D reconstruction, microstructure, mechanical properties, compression, finite element

## Abstract

Based on electron backscatter diffraction (EBSD), hollow structures of Ni foam struts fabricated by electroplating on a chemically removable template were observed. Three-dimensional (3D) pore structures of Ni foams were also obtained using X-ray computed tomography (CT), and microstructural features such as porosity, pore size and strut thickness were statistically quantified. Evolution of microstructure and mechanical properties during ex situ compression of open-cell Ni-foams was investigated based on X-ray CT, and experimental results were compared with predictions by the finite element method (FEM). 3D microstructures obtained by X-ray CT revealed that the stress drop started with the buckling of struts at the center of the Ni-foams. The flow stress increased after the buckling of the struts spreads to most of the regions. For effective simulation of the compressive deformation and determination of the microstructural evolution, small domains of interest were selected from the entire set of observed 3D microstructures based on X-ray CT, and struts of Ni foams with a hollow structure were simplified with relevant thin-solid struts. Numerical 3D modeling comprehensively disclosed that compression caused the transverse buckling of the struts, with the bending and buckling of struts thus reducing the stress. Thickness variation of the struts causes a change in the porosity of Ni-foams without a change in pore shape or connectivity. The overall range of strut thickness was from 59 to 133 μm, and the range of porosity values was from 80% to 93.7%. A stress drop was predicted with a decrease in the strut thickness or an increase in the porosity, as measured experimentally. It was also found that the stress drop contributed to an increase in the calculated energy absorption efficiency.

## 1. Introduction

Metal foams have an outstanding combination of mechanical properties, such as light weight, high strength-to-weight ratio, and good energy absorption capacity [1]. For this reason, they have been utilized in a variety of applications in many fields including the automobile, aerospace, and defense industries [2]. Many studies have been published on the compressive properties of metal foams. Three deformation stages, linear elastic, plateau, and densification, can be identified from the typical stress–strain curves of metal foams under compression [3]. The plateau stage with practically constant stress can be considered as an energy absorber zone, and the densification stage with a rapid increase in stress can be considered a safety backup zone [4].

Various investigations on testing and design of energy absorbers using metal-based foams with good energy absorption capacity have been reported. Kilicaslan et al. [5,6] studied the quasi-static and dynamic crushing responses of multi-layer trapezoidal aluminum corrugated sandwiches structures. Rem et al. [7] showed that the multi-layer aluminum foam sandwich structure has a much higher plateau stress than conventional bulky foam, and the stress oscillated during the plateau stage. Reglero et al. [8] concluded that the design and fabrication of an energy absorber prototype showed the potential of aluminum foam as an energy absorber material.

Numerous factors, such as material density, pore size, shape and connectivity, pore defects, and specimen size, affect mechanical properties of metal foams. With a decrease in foam density, the stress in the plateau stage is flattened and the energy absorption capacity is reduced [9]. In addition, the shape of pores is closely related to the collapse of the structures [10,11]. As pore size decreases, the Young’s modulus decreases, and the plateau stress increases [12]. Closed-cell metal foams exhibit a strain rate dependence in both absorption energy and plateau stress [13]. On the other hand, open-cell metal foams show a weak dependence of the yield strength and absorption energy on strain rate [14,15]. It is also known that regular pores improve mechanical properties of metal foams [3,16]. With more irregular pores, the reduction of Young’s modulus and shear modulus tend to be relatively large [17,18]. Greatly irregular foams have a larger tangential modulus at low strains and a lower stress at high strains than a more regular foam [19]. Furthermore, defects in pore structure contribute to a significant decrease in the mechanical properties, especially in small specimens. The partially coupled cells and missing cells caused critical variations in the stress–strain response [20]. Specimen size also affects mechanical properties of metal foams. The elastic modulus and yield strength of metal foams increase with the side length of samples [21]. Onck et al. [22,23] showed that the elastic modulus and plastic collapse strength of metal foams increase to a plateau level with an increase in the ratio of specimen size to cell size. Elevated temperature resulted in a decrease in stress and an increase in ductility of the cell walls [24,25].

Numerical approaches are an effective way to capture the relationship between microstructural features and mechanical properties of metal foams during deformation. Voronoi tessellation can be used for 3D model generation of virtual specimens with microstructural features. Many researchers have performed numerical modeling based on Voronoi models [12,17,18,21]. Kadkhodapour et al. [26] conducted a numerical analysis using FE modeling with various pores shapes, such as sphere, cubic, vertical elliptic, and horizontal elliptic cells.

Micro-CT is a powerful tool to characterize metal foam structure in a non-destructive manner. The initial microstructure and local deformation mechanisms of a polyurethane foam during compression were investigated by means of X-ray microtomography and finite element modeling [27]. The deformation behavior and fracture of an open-cell nickel foam were analyzed using synchrotron X-ray microtomography [28]. Compression tests were associated with strain localization phenomena due to the buckling of struts in a weaker region of the foam. Ramirez et al. [29] investigated mechanical behaviors during compression, and analyzed the cause of collapse of the structure based on micro-CT and FE modeling. Michailidis et al. [30] explained the deformation and buckling phenomena of struts and studied the effect of strain rate on the mechanical response of foams. As the pores collapse, the cell walls undergo bending, rotation, buckling, fracture, and tearing [31]. Moreover, deformation first occurs in the thin part of the cell walls, and then, parts of the weakest cell wall begin to collapse. This collapse process causes the structure to be densified. A serial sectioning method, which is one of the destructive methods, polishes and captures specimens at regular intervals and was carried out to analyze Ni foams [32,33,34]. As a result, it was confirmed that the local plastic deformation had a strong influence on the collapse mechanism of metal foams. Most studies on Ni foam modeling were focused on solid struts. Open-cell Ni foams with hollow struts can be easily manufactured by electroplating on a thermally or chemically removable template. A hollow pentagonal dodecahedron (HPD) model was proposed and recently investigated [35].

In the present research, evolution of microstructural features and mechanical properties during compression of open-cell Ni foams was examined in detail based on micro-CT and FE modeling. Hollow grain structures of Ni foam struts fabricated by electroplating on a chemically removable template were observed using electron backscatter diffraction (EBSD). In order to reflect the hollow struts, overall porosity or solid volume fraction of model domains was simply modified and verified based on predicted mechanical responses. Four different porosity models with a similar pore distribution were generated by adjustment of strut thickness and their compressive responses were examined using FEM. An ex situ compression test was also performed and the relationship between evolution of microstructure and mechanical properties was investigated. A small domain of interest was selected from the whole geometry of a compression sample, and numerical simulations were carried out on it to capture the evolution of microstructural features. The collapse mechanism of struts and flow behaviors including stress drop have been explored both experimentally and numerically. Variation of the energy absorption efficiency was calculated and compared, based on experimental and predicted flow curves.

## 2. Experimental and Numerical Procedures

### 2.1. Materials

Open-cell Ni foams fabricated by electroless-electroplating on chemically removable polymer templates were used for compression tests. Disc-shaped compression specimens were 10 mm in diameter, and the overall thicknesses of three different specimens were approximately 2.05 mm (Specimen 1), 1.05 mm (Specimen 2), and 1.03 mm (Specimen 3), respectively. When cutting the Ni foams, wire electrical discharge machining (EDM, FANUC ROBOCUT) was utilized to minimize specimen deformation.

### 2.2. Compression Tests

Compression tests were carried out on discs using a testing machine (INSTRON 5982) with a 2 kN load cell at room temperature. The cross-head speed during compression was 0.1 mm/min. Specimens were placed between two parallel plates of the testing machine, and compression proceeded as the top plate descended to the bottom plate. An ex situ compression test was also performed to investigate the microstructure evolution and mechanical behaviors in detail. When the compression specimen reached the targeted strain, it was moved into the micro-CT repeatedly. Initial and deformed microstructures at three different strains were measured using micro-CT.

Strain was obtained based on displacement. An extensometer or a strain gauge is required to accurately measure the strain during a compression test. However, due to the structural weakness and thin geometry of Ni foams, it is difficult to measure the specimen strain with a strain gauge. Notably, Kalidindi et al. [36] investigated the machine compliance to correct flow curves, which were obtained from loads and displacements of a testing machine without a strain gauge. For low compressive loads associated with foam materials, displacement correction based on machine compliance was negligible, considering the total displacement [23]. Thus, machine compliance was not considered in this study.

### 2.3. Microstructure Characterization

The microstructure and orientations of the Ni foams were also measured by means of automated electron backscatter diffraction (EBSD) (AZtec, Oxford) in conjunction with high-resolution scanning electron microscopy (HR-SEM, Jeol7001F). Ni foams were mounted using an electro-conductive cold-mounting resin (Technovit 5000), and they were mechanically polished using silicon carbide abrasive discs up to 1500 grit. To reduce the deformation layer caused by mechanical grinding from the surface, a 1μm diamond suspension was used for finer polishing. Final auto-polishing was carried out using a colloidal silica suspension for approximately 20 min at 13 N. Triclinic sample symmetry was applied to create pole figures from the EBSD data. The mapping step size was 0.5 μm.

### 2.4. 3D Reconstruction

Ni foams were imaged via non-destructive X-ray computed micro-tomography (Nikon XT H 160 Micro-CT). Conditions of the X-ray source were set to 155 kV for beam energy and 157 μA for beam current. Specimens were placed on a motor controlled rotating stage, and X-ray projections were taken from several angles, resulting in 1440 projections. The beam hardening and noise values were adjusted, and unnecessary parts were removed and leveled from the whole geometry by setting the regions of interest (ROI). Stacked images were rendered into an appropriate volume domain using a three-dimensional visualization package (AVIZO). Surface shape information was generated in the STL format from the rendered volume domain. The surface shape information was eventually utilized for discretization of Ni foams consisting of volume elements. Reconstruction processes of 3D geometry from stacked images obtained from X-ray CT are graphically summarized in the supplementary part.

### 2.5. Numerical Procedure

Mechanical responses and microstructural evolution during compression of open-cell Ni foams were analyzed using finite element approaches (DEFORM3D). 3D volume meshes consisting of 4-node tetrahedron elements were used to discretize reconstructed 3D models. Considering the simulation efficiency, reduced 2 mm × 2 mm × 2 mm cubic domains taken from the whole disc with a diameter of 10 mm were selected and discretized into microstructure models.

Material properties of pure Ni were assigned to the Ni foam models. Materials constants and modeling parameters are summarized in Table 1. An interpolated flow curve obtained from a previous study was applied for plastic responses [37]. The top and bottom molds were set to be rigid. The top mold was moved in the direction of the bottom, and the step increment was set to be 0.001 mm/step. The conjugate gradient solver with the direct iteration method was selected [38]. Automatic remeshing was used to facilitate convergence [39].

## 3. Results and Discussion

### 3.1. Cross Section of a Ni Strut

**Figure 1:**
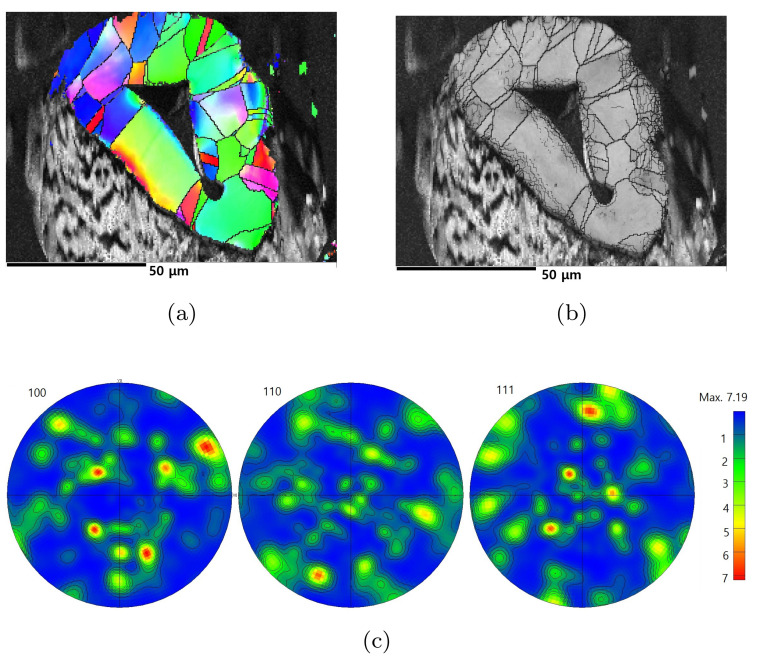
Electron microscope images of the cross section of a strut of Specimen 1: (**a**) inverse pole figure (IPF) map, (**b**) image quality (IQ) map, and (**c**) pole figures (PFs). Contours of pole figures: 0.65, 1.05, 1.45, 1.85, 2.25, 2.65.

Figure 1 illustrates the typical grain structure and crystallographic texture obtained from the cross section of a strut of Ni foams (Specimen 1) using scanning electron microscopy (SEM) and electron backscatter diffraction (EBSD). Figure 1a,b shows an inverse pole figure (IPF) map and image quality obtained from EBSD. Grain identification angles are specified with thin and thick lines corresponding to $2^{\circ }$ and $15^{\circ }$, respectively. Based on IPF maps, it is found that struts usually possess a hollow structure in the center. The wall of the hollow structure consists of a few grains through the thickness direction, and its thickness is approximately 20 μm. The computed pole figures of (100), (110), and (111) from EBSD data reveal a random distribution, as shown in Figure 1c.

### 3.2. Reconstructed Ni foams

**Figure 2:**
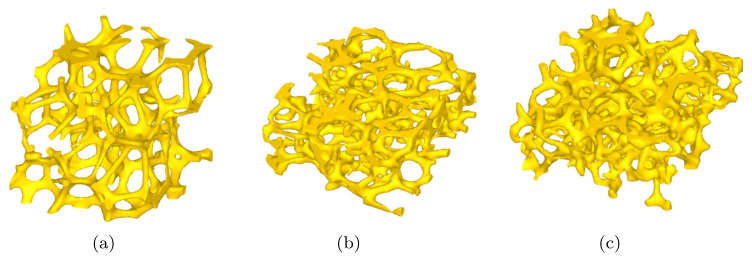
Reconstructed 3D microstructures with various pore structures. (**a**) Specimen 1 (2.05 mm high), (**b**) Specimen 2 (1.05 mm high), and (**c**) Specimen 3 (1.03 mm high).

Figure 2 illustrates three different microstructures of open-cell Ni foams used for uniaxial compression. The microstructures were obtained from micro-CT followed by appropriate reconstruction. Each microstructure model possesses different pore morphologies and porosities. Microstructure of metal foams consists of solid struts and empty pores.

For a numerical analysis, reconstructed microstructure based on X-ray CT was discretized. In fact, it would be most reasonable to directly model the hollow grain structure observed in Figure 1. For 3D reconstruction based on X-ray CT, the hollow strut of the Ni foams is still difficult to handle. Instead, the solid strut thickness was adjusted to model the microstructure. The solid volume of model domains was decreased, or the porosity was increased, considering the hollow structure inside struts. The adjusted model domains were verified based on predicted mechanical responses.

Various microstructural features including porosity, average strut thickness, and slenderness ratio are summarized in Table 2. Dimensions of the 3D microstructures are specified in detail. The high porosity model possesses a relatively high slenderness ratio, compared with the other models with low porosities. The latter models possess thicker and shorter struts than the former. From the structural view point, the slenderness ratio is a measure of the propensity of a column or strut to buckle. Specimen 1 possesses the greatest average slenderness ratio of 27 among all specimens, and thus strut buckling is more highly expected in this specimen. The slenderness ratio λ is given by the following equations [40]:

(1) $$\lambda =\frac{l}{r}$$

(2) $$r=\sqrt{\frac{I}{A}}$$

where *l*, *r*, *I*, and *A* are the length, radius of gyration, second moment of the area, and cross-sectional area, respectively. High slenderness structures or thin struts result in easier buckling than low slenderness or thick strut structures.

**Figure 3:**
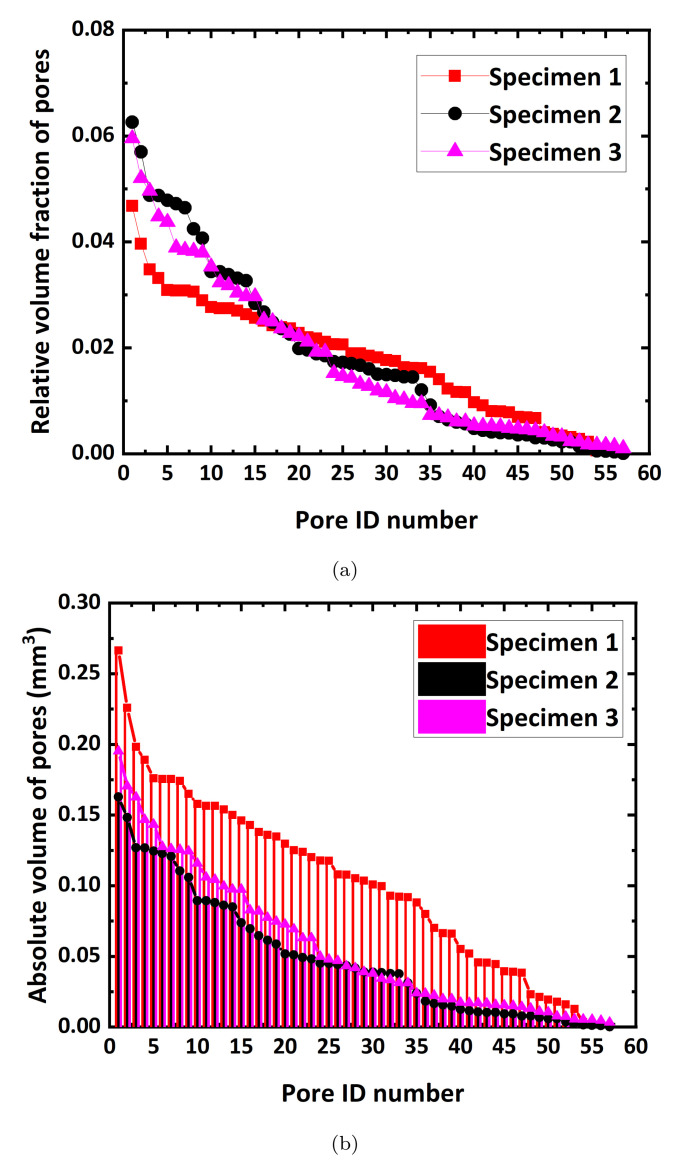
(**a**) Relative volume fraction (RVF) of pores, and (**b**) absolute volume (AV) of pores in the microstructures, as shown in Figure 2.

Various features of pores, such as pore size, regularity, morphology, and connectivity affect the mechanism of pore collapse and the mechanical properties of metal foams. The regularity of pores can be defined as given in Equations (3) and (4):

(3) $${\left(RVF\right)}_{i}=\frac{{\left(AV\right)}_{i}}{\sum_{i=1}^{n}{\left(AV\right)}_{i}}$$

(4) $$\sum_{i=1}^{n}{\left(RVF\right)}_{i}=1$$

where AV is the absolute volume of pores, and RVF is the relative volume fraction of pores in the model. Distribution features of RVF and AV in the three different models shown in Figure 2 are presented in Figure 3. When pore distributions inside the models have a similar regularity, the RVF distributions of pores closely coincide, and the AV distributions also display a similar trend. Different distributions of RVFs and AVs of pores usually result in different mechanical responses.

**Figure 4:**
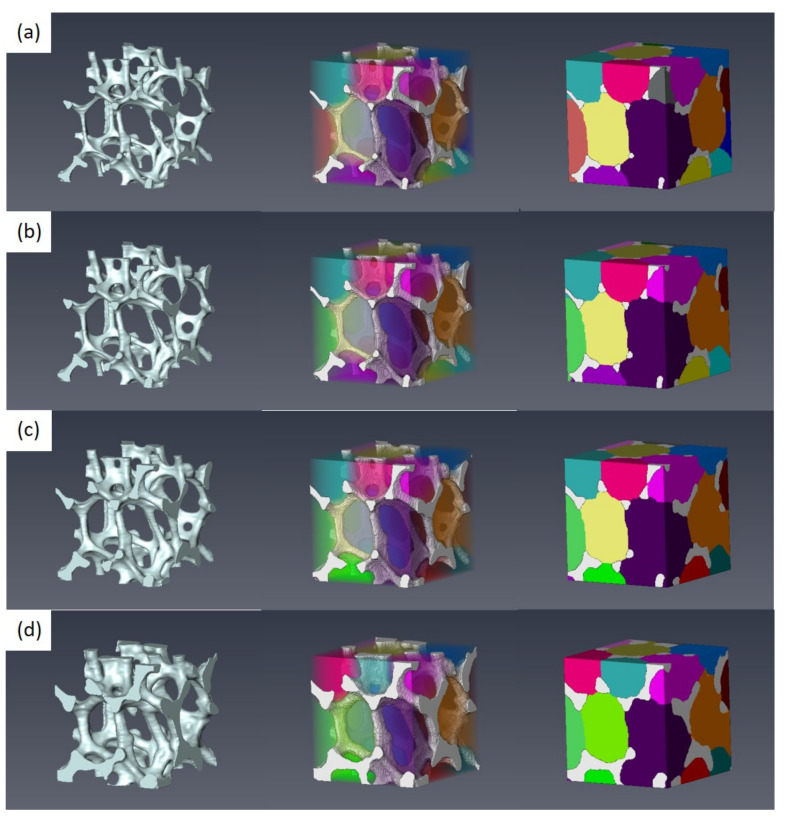
Four different model domains with different strut thickness: (**a**) thinner, (**b**) standard, (**c**) thicker, and (**d**) the thickest models.

Numerous factors affect the mechanical properties of metal foams, such as porosity, pore size, pore shape, pore connectivity, etc. It is difficult to experimentally control each microstructural feature individually. A virtual specimen based on the model domain obtained from micro-CT was utilized to easily control the microstructural features mentioned above. Three different models were generated through control of the strut thickness, using a part of Specimen 1 (standard model). The three models possess thinner, thicker and the thickest struts, compared to the starting standard model. All four models created are presented in Figure 4. The starting standard model is given in Figure 4b, and the other three models with thinner, thicker, and the thickest struts are presented in Figure 4a,c,d, respectively. The morphological shape and connectivity of pores are similar to each other. Table 3 summarizes microstructural features of four different model domains. With an increase in the average strut thickness, both the porosity and average slenderness ratio decrease.

**Figure 5:**
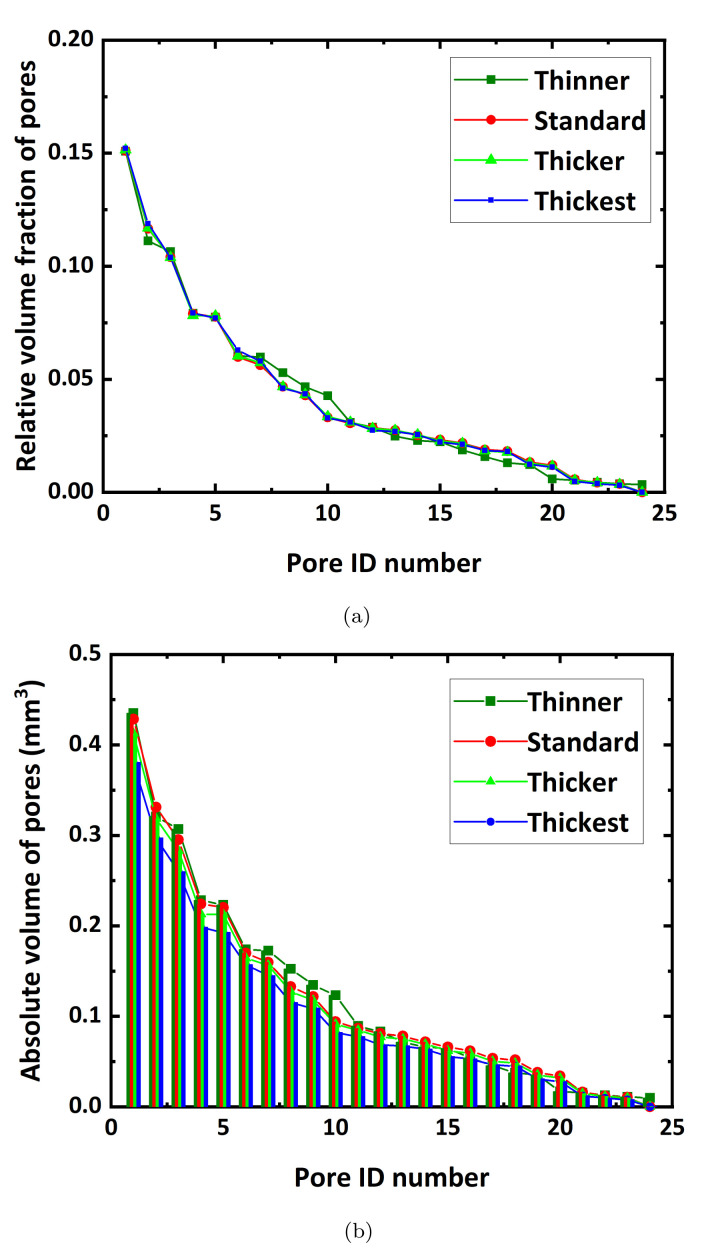
(**a**) The relative volume fraction (RVF) of pores, and (**b**) absolute volume (AV) of pores in various model domains with different strut thickness, as shown in Figure 4.

Figure 5 shows distributions of the RVFs and AVs of pores in descending order of size inside the four different strut models. The RVF distributions of pores for the four models are almost identical. In addition, the AVs of pores reveal a constant decreasing rate as the struts become thicker. This indicates that these models differ only in pore volume fractions, and the morphology, number, and connectivity of pores are constant.

### 3.3. Modeling Uniaxial Compression

**Figure 6:**
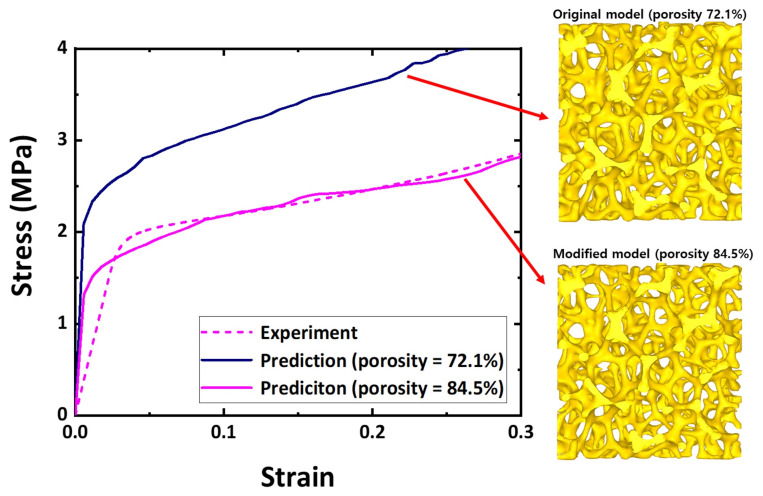
Effect of porosity change on predicted flow behaviors of Specimen 3 during compression.

Microstructure obtained from X-ray CT underestimated the porosity of the model domains because the hollow structure in the center of the struts was not considered. Figure 6 illustrates two different microstructures of Specimen 3 with different porosities, and their predicted strain–stress distributions were compared to an experimental result. Each microstructure model for Specimen 3 was generated in a similar manner, as discussed in Figure 4, and thus, they possessed a similar pore distribution. The original microstructure model with a porosity of 72.1% overestimated the flow behavior, compared to the experimental result. The flow behavior of the modified model with increased porosity of 84.5% closely matched that of the experiment. Porosity modification to reflect the hollow structure of the struts thus appears to be reasonable. The original microstructure domain was selected to reasonably represent pore and strut distributions of the Ni foams.

**Figure 7:**
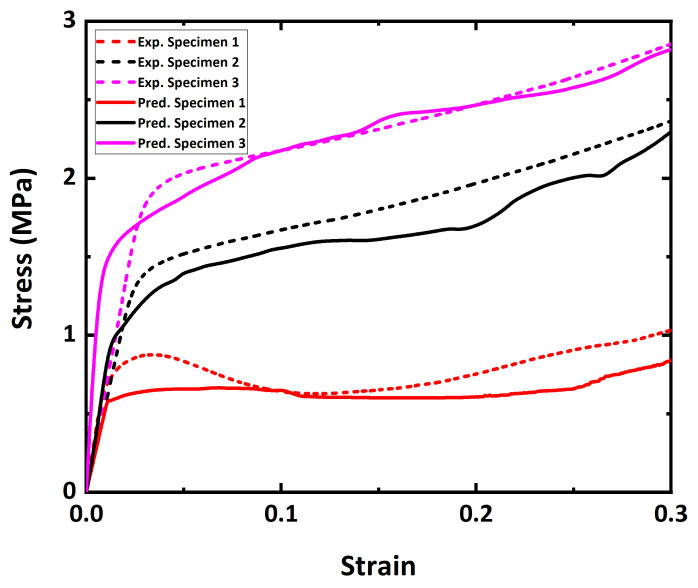
Comparison of stress–strain curves between experiments and predictions of various Ni foams.

Both the predicted and measured flow curves during compression of Specimens 1, 2, and 3 are presented in Figure 7. Overall trends of the flow curves for both experiments and predictions revealed similar behaviors. The stress–strain curves illustrate a typical trend of mechanical behaviors of metal foams, and three deformation stages are confirmed, i.e., linear elastic, quasi-plateau, and densification stages. Each stage approximately corresponds to strain ranges of 0 to 0.03, 0.03 to 0.25, and greater than 0.25, respectively. Energy absorption dominantly occurs in the quasi-plateau stage with nearly constant stress. During densification, compressive stresses quickly increase. In the densification stage of the flow curves, metal foam structures collapse, and pores are shrunk.

Based on the flow curves, it is found that the flow stress of Ni foams increases as the porosity level decreases. Porosity is the most dominant factor determining the flow stress. Ni foams with low porosities (Specimens 2 and 3) show a gradual increase in the quasi-plateau stage. The plateau stress of Ni foams with a greater porosity (Specimen 1) remains relatively low, compared to those of Ni foams with low porosities. A stress drop (SD) distinctly occurs at strains between 0.03 and 0.15 in the experimental flow curve of Specimen 1 with a porosity of 92.2%. Note that this is not observed in other specimens with low porosities of 86.1% and 84.5%. In the predicted flow curve of Specimen 1, a slight stress drop is observed in the quasi-plateau stage. The stress drop will be further discussed later based on ex situ compression.

**Figure 8:**
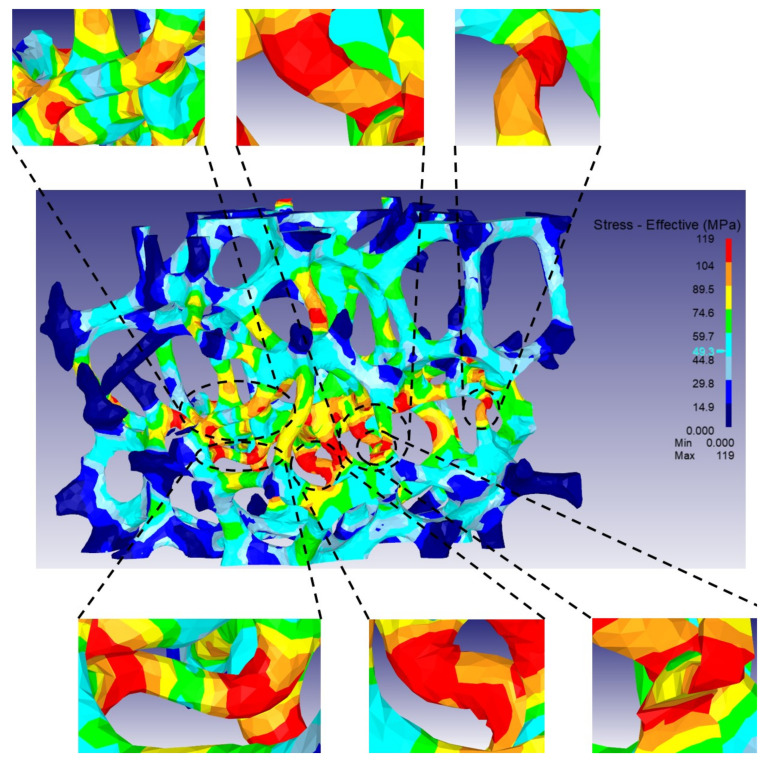
Distribution of effective stress for 3D model domain of Specimen 1 at a strain of 0.15.

Figure 8 shows stress distributions at the end of the stress drop (ϵ = 0.15), which is computed from the microstructural model of Specimen 1 with a porosity of 92.2%. Similar to the experimental behavior, various struts in the center of the model are buckled along the vertical direction during compression. The struts with buckling usually possess high stress values.

### 3.4. Ex Situ Compression

**Figure 9:**
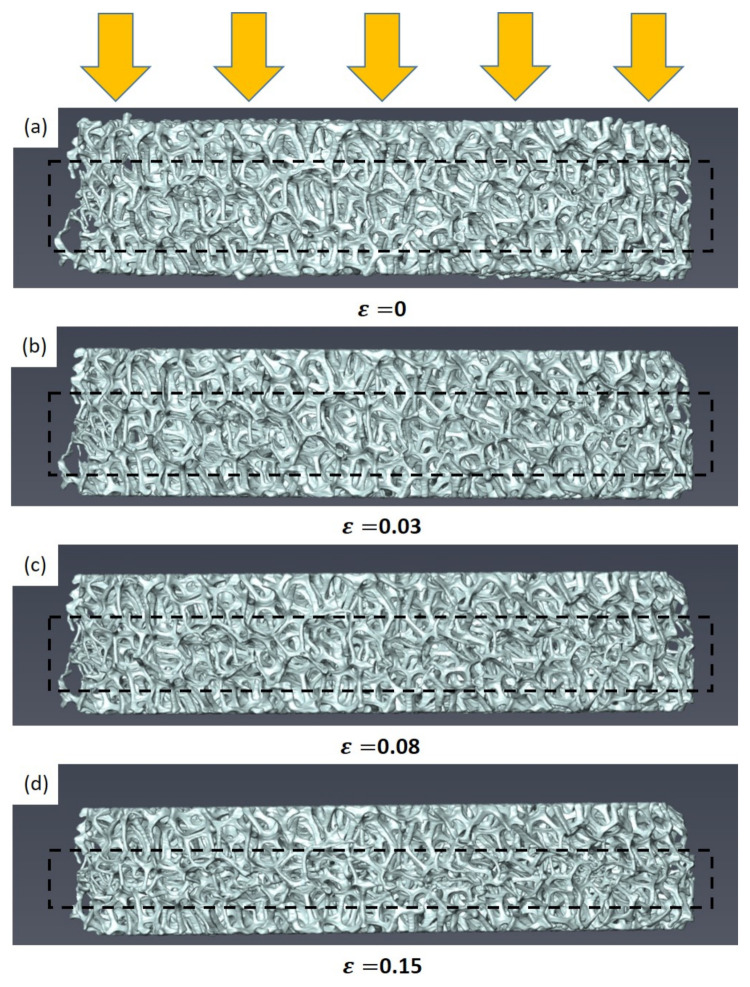
Observed microstructural evolution of the whole disc with strain during ex situ compression.

An ex situ compression test was conducted to further analyze the relationship between compressive flow behaviors and microstructure evolution of Specimen 1. Specimen 1 with a high porosity of 92.2% revealed a stress drop during compression, as shown in Figure 7. Microstructure evolution of the whole disc with an increase in compressive strain is presented in Figure 9. The whole disc geometry with a diameter of 10 mm was measured using micro-CT during ex situ compression. Figure 9a illustrates initial state of open-cell Ni foams, and Figure 9b–d are microstructures obtained at strain values of 0.03, 0.08, and 0.15, respectively. In particular, Figure 9c is a deformed microstructure corresponding to a stress drop. The observed microstructure accurately demonstrated the collapse mechanism of open-cell Ni foams. Buckling occurred in the struts near the center region of the specimen. Figure 9d is the microstructure corresponding to a return to an increase in compressive stress. It is also observed that many struts were compressed after the stress drop.

**Figure 10:**
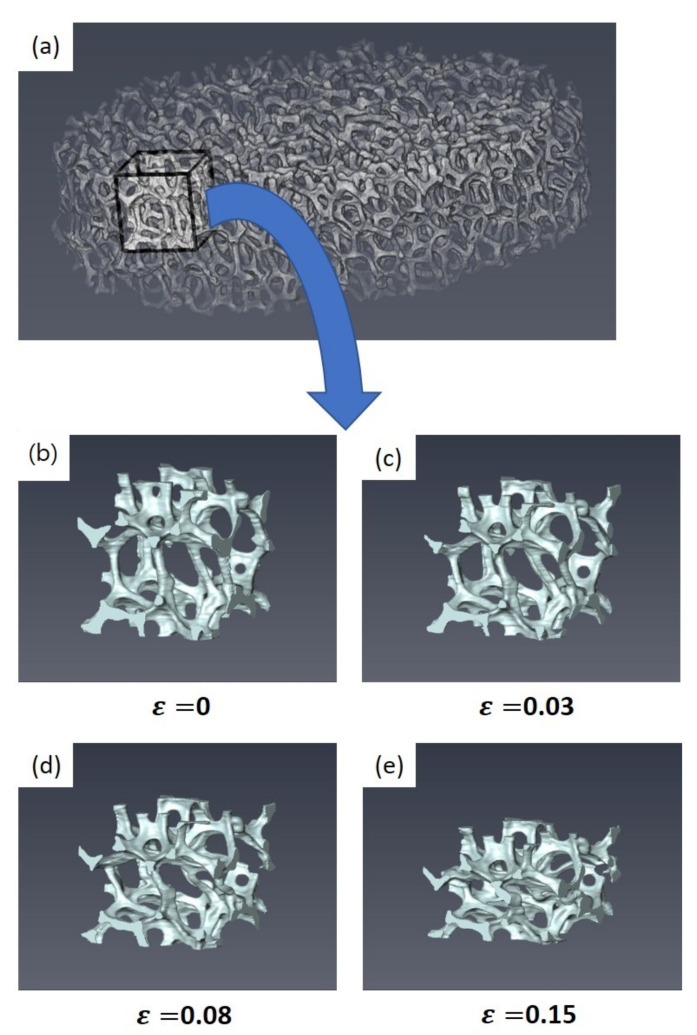
Observed microstructural evolution of the selected geometry of interest with strain during ex situ compression.

Figure 10 shows the observed microstructural evolution of a selected small geometry of interest to trace bending and buckling of Specimen 1 in detail. Notably, the micro-bending and buckling mechanism of the open-cell Ni foams was identified using the small geometry of interest. The struts aligning with the compression direction were bent and buckled during compression. With an increase in strain, struts inside the small geometry of interest were bent and squeezed. Beyond a strain of 0.15, dense collapse of struts was expected with an increase in strain.

**Figure 11:**
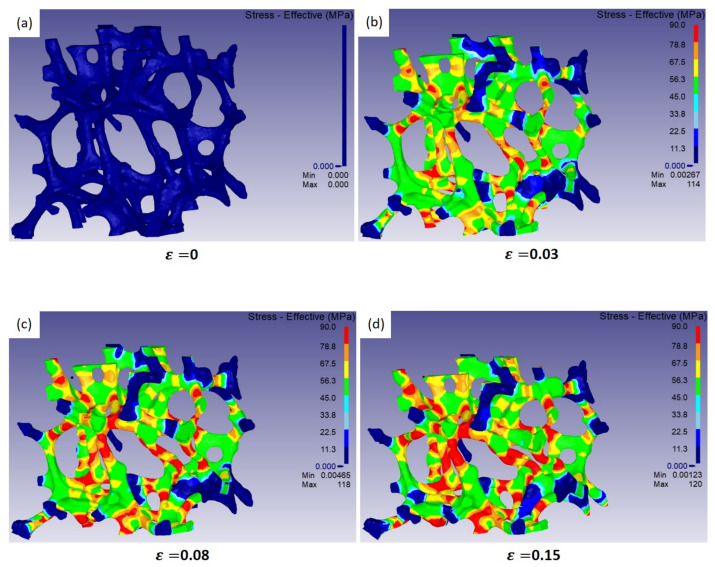
Predicted microstructural evolution of the selected model domain of interest with strain.

Using the small domain of interest, as shown in Figure 10a, compression processes were numerically simulated under non-periodic boundary conditions. Figure 11 illustrates the predicted microstructure evolutions with strain during compression. In the model domain, struts aligning with the compression direction experienced high stress. Most bending and buckling was observed in those struts with high stress levels. Overall, the collapse mechanism of struts predicted by the model domain is similar to that observed by experiments, as shown in Figure 10.

**Figure 12:**
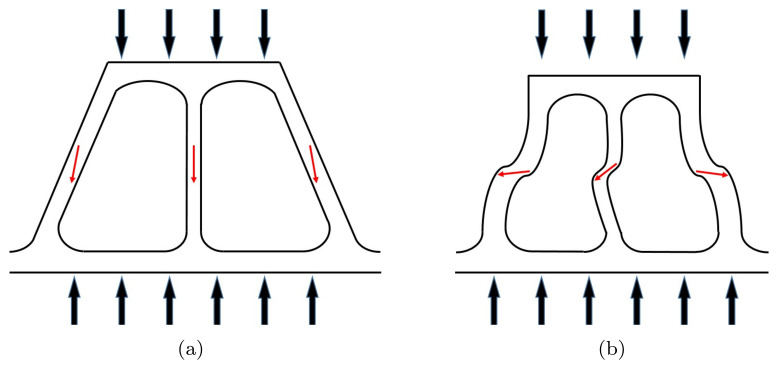
Schematic diagrams of struts under compressive loading: (**a**) a initial state and (**b**) a deformed state.

Combined approaches using X-ray CT and FEM are useful for evaluating compressive mechanical responses of foam materials. Here, bending and bucking of struts of Ni foams are simply illustrated, based on experiments and numerical predictions. Figure 12 is a schematic diagram that presents the deformation procedure of structural struts during compression. Structural struts aligning with the loading direction mainly experience and accommodate most external loading. In particular, the regions with red arrows frequently are bent under compressive loading. When bending occurs in the transverse direction, as specified with red arrows, a stress drop occurs. This bending mechanism is more likely to occur when the slenderness ratio increases.

### 3.5. Effect of Strut Thickness on Bending and Buckling

From a previous study on ex situ compression, it is found that strut thickness, or slenderness, is the dominant factor affecting the stress drop of Ni foams. Here, the effect of strut thickness on mechanical properties and microstructure evolution was investigated based on various model domains, as illustrated in Figure 4. When considering the slenderness ratio, the model domain with thinner struts corresponds to the greatest slenderness ratio, and the last model with the thickest struts is equivalent to the smallest slenderness ratio.

**Figure 13:**
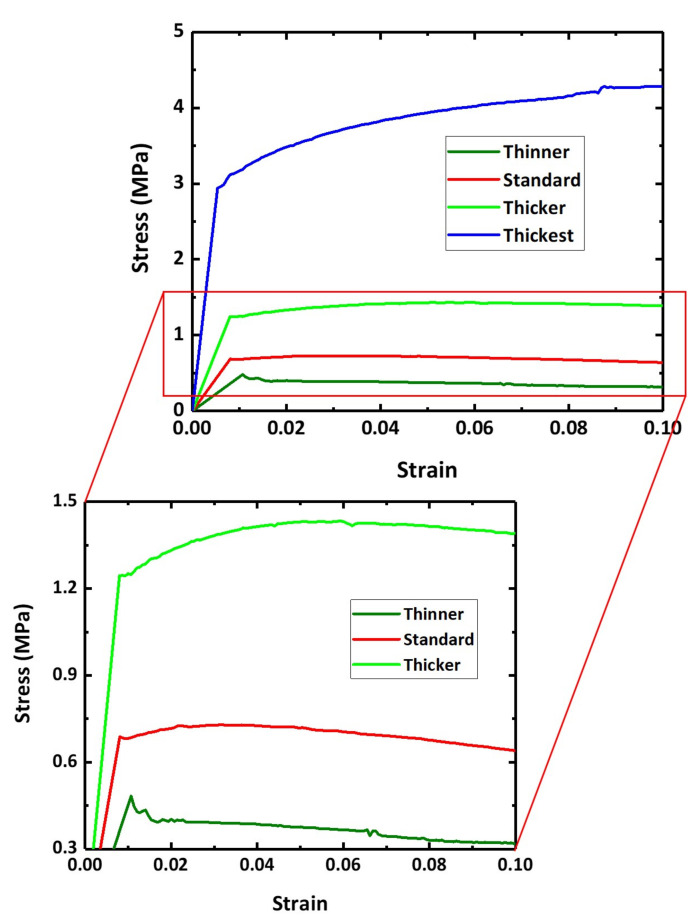
Predicted flow curves of various model domains with different strut thickness.

The predicted stress–strain curves of each model obtained by FE simulation are displayed in Figure 13. A stress drop does not occur in the thickest model. The other three models with thinner, standard, and thicker struts reveal minor stress drops. The degree of the stress drop increases with a decrease in thickness of the struts, and the stress drop occurs when the struts are thin. Note that the thinner the struts are, the higher the slenderness ratio is.

**Figure 14:**
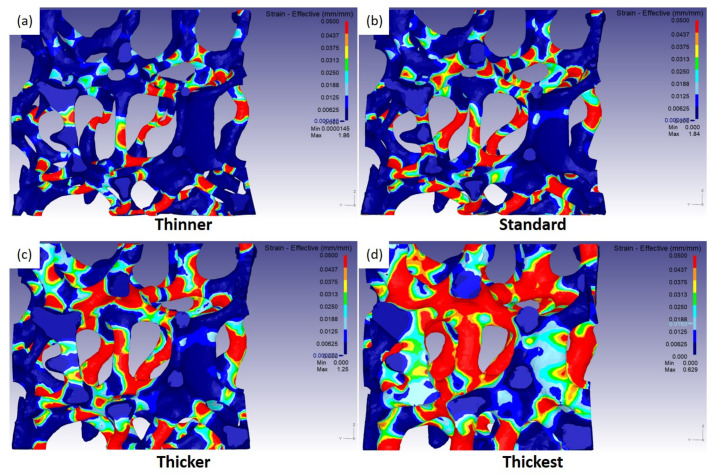
Distribution of effective strain of various model domains with different strut thickness at a macroscopic strain of 0.1: (**a**) thinner, (**b**) standard, (**c**) thicker, and (**d**) the thickest models.

Figure 14 shows the effective strain distribution of four different models at a strain of 0.1. The effective strain was defined as $\sqrt{2/3\varepsilon_{ij}\varepsilon_{ij}}$, where ε is a strain tensor. In the thinner model, as shown in Figure 14a, large effective strains are mainly observed in the middle of vertical struts. This implies that bending and buckling occur there. With an increase in struts thickness, the regions with large effective strains are gradually enlarged. In the thickest model, as shown in Figure 14d, considerably large regions reveal large effective strains. Wide regions accommodate external strain together, and thus little buckling occurs. From the view-point of the structural slenderness ratio, it can be understood that a large slenderness ratio easily results in buckling, and a small ratio rarely causes buckling.

### 3.6. Energy Absorption

One of the outstanding advantages of metal foam construction is a high strength-to-weight ratio. From a previous study, energy absorption and ideal energy absorption efficiency have been defined as follows [13]:

(5) $$W_{v}=\int_{0}^{\varepsilon }\sigma d\varepsilon$$

(6) $$I=\frac{\int_{0}^{\varepsilon }\sigma d\varepsilon }{\sigma \cdot \varepsilon }$$

where $W_{v}$ is the energy absorption per unit volume, and *I* is the ideal energy absorption efficiency, respectively. The energy absorption and ideal energy absorption efficiency can be evaluated by integrating the area under the flow curve. These equations were used to study the relationship between energy absorption (efficiency) and stress drop.

**Figure 15:**
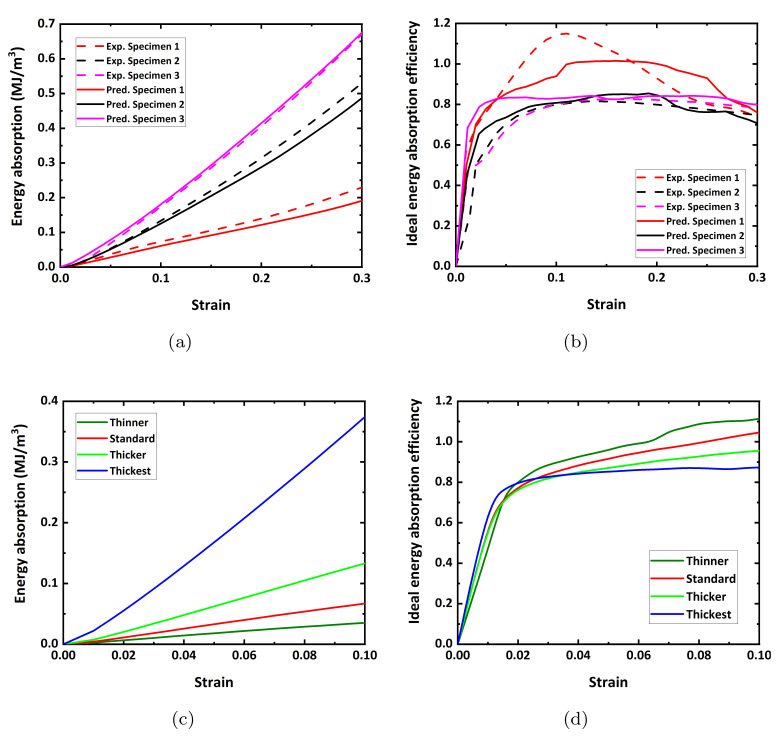
(**a**) Energy absorption and (**b**) ideal energy absorption efficiency of experiments and predictions according to different porosity. (**c**) Energy absorption and (**d**) ideal energy absorption efficiency of various model domains with different strut thickness.

Energy absorption and ideal energy absorption efficiency of various Ni foams are given in Figure 15. The results computed from experimental and predicted flow curves are presented in Figure 15a,b, respectively. Figure 15c,d are the results computed from various flow curves of model domains with different strut thickness, as shown in Figure 13. As shown in Figure 15a,c, the energy absorption simply increases as the porosity increases. Interestingly, this is not the case for the ideal energy absorption efficiency. In Figure 15b,d, the ideal energy absorption efficiency increases when a stress drop occurs. Without the stress drop, however, the ideal energy absorption efficiency showed little change, indicating that it is insensitive to porosity change. Overall, it can be concluded that the stress drop improves the energy absorption efficiency, demonstrating that our approach is an appropriate design strategy of light-weight construction using metal foams.

## 4. Conclusions

Evolution of microstructure and mechanical properties during compression of open-cell Ni foams with hollow struts was analyzed through experimental and numerical approaches based on micro-CT and FEM.

1. Based on electron backscatter diffraction (EBSD), it was found that Ni foam struts consisted of a few single grains along the thickness direction. Crystallographic orientations of grains showed a random distribution. The effects of the unique grain structure and random orientations on mechanical responses require further studies.
2. Reconstructed 3D microstructural models modified to indirectly reflect the hollow structure were used for a compression simulation, and the predicted flow behaviors were compared with the experimental results. Flow curves of Ni-foams consisted of linear elastic, quasi-plateau, and densification stages. A stress drop at the beginning of the flow curve was associated with high porosity.
3. During ex situ compression, the relationship between microstructural evolution and buckling of struts was analyzed in detail. The bending and buckling of strut columns along the transverse direction mainly caused the stress drop.
4. The effect of strut thickness on compressive flow behaviors and microstructural evolution was also examined using various model domains, adjusting the strut thickness only. Predicted flow curves revealed that a stress drop occurred in all models with an average slenderness ratio greater than approximately 10. As the strut thickness becomes thinner, the amount of the stress drop increases.
5. Energy absorption generally increased with a decrease in porosity, but the ideal energy absorption efficiency showed insensitivity to porosity. The ideal energy absorption efficiency increased with a drop in stress. This indicates that metal foams possess an outstanding absorption-to-weight ratio in terms of use as structural materials.

## Figures and Tables

**Table 1:** Summary of materials constants and friction coefficient.

Young’s Modulus E	Poisson Ratio	Friction Coefficient
60 GPa	0.3	0.23

**Table 2:** Summary of microstructural features of various open-cell Ni foams, as shown in Figure 2.

	Specimen 1	Specimen 2	Specimen 3
Dimension [mm]	2.01 × 2.01 × 2.05	2.09 × 2.12 × 1.05	1.96 × 2.09 × 1.03
Porosity (%)	92.2	86.1	84.5
Number of elements	144,367	233,878	245,226
Mean strut thickness [μm]	71.6	96.4	81.8
Mean slenderness ratio	27.0	6.7	9.4

**Table 3:** Summary of microstructural features of various model domains with different strut thickness, as shown in Figure 4.

	Thinner	Standard	Thicker	Thickest
Porosity (%)	93.7	92.2	88.6	80.0
Number of elements	126,807	158,742	183,823	261,050
Mean strut thickness [μm]	59.2	69.0	94.2	133.0
Mean slenderness ratio	27.3	20.8	14.9	9.9
